# Methods underpinning national clinical guidelines for hypertension: describing the evidence shortfall

**DOI:** 10.1186/1472-6963-6-47

**Published:** 2006-04-05

**Authors:** Fiona Campbell, Heather O Dickinson, Julia VF Cook, Fiona R Beyer, Martin Eccles, James M Mason

**Affiliations:** 1 University of Newcastle upon Tyne, Centre for Health Services Research, 21 Claremont Place, Newcastle upon Tyne, NE2 4AA, UK; 2 University of Durham, School for Health, Wolfson Research Institute, Queen's Campus, University Boulevard, Stockton-on-Tees, TS17 6BH, UK

## Abstract

**Background:**

To be useful, clinical practice guidelines need to be evidence based; otherwise they will not achieve the validity, reliability and credibility required for implementation.

**Methods:**

This paper compares the methods used in gathering, analysing and linking of evidence to guideline recommendations in ten current hypertension guidelines.

**Results:**

It found several guidelines had failed to implement methods of searching for the relevant literature, critical analysis and linking to recommendations that minimise the risk of bias in the interpretation of research evidence. The more rigorous guidelines showed discrepancies in recommendations and grading that reflected different approaches to the use of evidence in guideline development.

**Conclusion:**

Clinical practice guidelines as a methodology are clearly still an evolving health care technology.

## Background

Clinical practice guidelines can provide building blocks for changing and improving health care [1] and are a useful means of bridging the gap between scientific research evidence and usual practice [2]. They are defined as 'systematically developed statements to assist physicians and patients about appropriate health care for specific clinical circumstances' [3]. To achieve their potential as effective tools for improving health care they need to maximise their validity, a feature related to the use of evidence within a guideline and development using a multidisciplinary process [4]. However, despite an apparently explicit methodology there are variations in what guidelines say and how they relate this to underlying evidence [3,5,6]. There is also concern that guideline development may be subject to external influence [7,8].

Like many other conditions hypertension has been the subject of many different international guidelines. The World Health Organisation (WHO) have described hypertension – defined as a blood pressure of greater than 140/90 mmHg – as one of the ten leading risk factors influencing the global burden of disease [9]. It is a contributory factor in ischaemic heart disease and cerebrovascular disease accounting for 20% and 10% of all deaths in England and Wales respectively [10]. Reducing blood pressure levels leads to significant reductions in cardiovascular and cerebrovascular morbidity and mortality [11]. Nevertheless the management of hypertension remains suboptimal: for example, 40% of the adult population of England suffer from hypertension, but current levels of detection and treatment result in only 9% of sufferers having their hypertension controlled to <140/90 mm Hg [12]. There is a clear need to improve the management of hypertension both in this country and worldwide. If guidelines, however, are to play a role in this improvement they will need to maximise their validity.

The aim of this study was to review how well 10 guidelines for hypertension addressed validity in terms of their methods and their use of published evidence.

## Methods

### Contributing guidelines

We reviewed the methods used in development and the key recommendations of ten current guidelines (see table 1) meeting the following criteria: they concerned the general management of hypertension, or the management of hypertension in specific populations; published in English and nationally or internationally recognised. Guidelines developed before 1994 were also excluded as they predated the publication and wide dissemination of the work by Field and Lohr which offered the first and seminal work on guideline methodology [1].

We used five guidelines (CMA [13], WHO [14], VHA [15], ICSI [16], SA [17]) meeting these criteria retrieved by the comprehensive search strategy employed by the German Guideline Clearing Report [7], whose search strategy covered 1990–1999. We updated the strategy by searching MEDLINE, EMBASE and OMNI from 1999 onwards using the thesaurus heading HYPERTENSION and limiting to guidelines or practice guidelines in English. This retrieved a further five guidelines (SIGN [18], ESH [19], JNC [20], BHS [21], NICE [22]) for consideration.

### Evaluation of guideline development methods

We evaluated the methods used to develop each guideline with particular reference to three dimensions that relate to the use of research evidence, as found in the full published report of each guideline:

• the construction of the guideline development group and its component stakeholders.

• the use of published literature and the strategy used in screening for the primary evidence; in particular, the use of existing systematic reviews or the performance of a new systematic review explicitly to answer questions posed by the guideline.

• the grading of evidence and recommendations: in particular, an explicit link between recommendations and supporting evidence.

### Evaluation of recommendations and their underlying evidence

We compared recommendations on four areas that were common to all the guidelines: diagnosis of hypertension, lifestyle modification, criteria for initiation of antihypertensive drug therapies and initial recommended drug therapy. We also explored links between recommendation grades and citations and looked at how these differed in recommendations for drug therapy and salt intake.

## Results

### Methods used to develop the guidelines

The measures used to assess the guideline development process are summarised in table 2.

Only three guidelines were constructed by multidisciplinary groups where the members' affiliations and conflicts of interest were described; these three guideline groups included patient representatives as well as key professional stakeholders. A further six guidelines provided only a list of names and institutional affiliations of members of the guideline development group. One further guideline gave no details of the guideline development group (see table 3).

Only one guideline conducted new systematic reviews to inform recommendations. Seven guidelines made extensive use of existing systematic reviews; three of these guidelines also stated that a search strategy based on MESH search terms was used for identifying relevant research evidence. A further two guidelines made limited use of existing systematic reviews.

There were different approaches used in the guidelines to assess the evidence available upon which to base a recommendation and upon the grading of the recommendation itself (see table 3 for a description of the grading systems used in the guidelines). Three guidelines did not grade either the evidence they cited or their recommendations. Two coded the evidence on the basis of study design but did not link this to the recommendations. In contrast, five guidelines graded the evidence and explicitly linked this to the recommendations. However, there were differences also between these grading systems with different criteria used to assess the contributing studies. The grading systems used by two guidelines (NICE, CMA) allowed for the quality of relevant randomised controlled trials and meta-analyses and the strength of their results to be analysed. The grading systems used by other guidelines did not allow for this more sensitive assessment of the evidence. Differences and shortcomings in these grading systems can be confusing and impede effective communication [23]. The GRADE system was developed as a result of these shortcomings and recommends an approach which takes into account study design, quality, consistency and directness in judging the considers the benefit harm ratio, quality of evidence, applicability, and baseline risk when translating to recommendation [23].

### Recommendations made by the guidelines

#### Diagnosis of hypertension

The guidelines were consistent in defining the threshold for hypertension as 140/90 mmHg and all agreed that blood pressure should be measured twice in a consultation on at least two separate occasions. The need for a full medical examination, clinical history and accurate blood pressure measurement was described in all of the guidelines. However, they differed on the recommended routine tests. All agreed that an electrocardiogram, blood chemistry, a complete blood cell count and urinalysis should be conducted during the initial assessment of hypertension, to assess broader cardiovascular risk. There was less agreement on the assessment of: total cholesterol, lipid profile, blood glucose, creatinine, blood calcium, thyroid stimulating hormone, gammaglutamyl transpeptidase and serum urate.

#### Lifestyle modifications

All of the guidelines addressed lifestyle modification as an integral part of the management of hypertension and as a first line treatment in mild hypertension, and made similar recommendations for weight loss, limiting alcohol and sodium intake, regular exercise and smoking cessation (see table 4). Guidelines typically recommended a target BMI of 18.5 – 25 kg/m^2^, restriction of salt intake to under 6 g/day and restriction of alcohol to 14 to 20 g (ethanol) per day for women and from 24 to 30 g (ethanol) per day for men. Differences in the daily limits for alcohol consumption may reflect the variations on guidance for sensible drinking in different countries. Most guidelines recommended a diet rich in fruit and vegetables with reduced saturated and total fats. Guidelines typically recommended 30–45 minutes of aerobic exercise three to five times per week. Although their recommendations were similar, guidelines lacked consistency in the estimations of the effect of lifestyle changes on blood pressure, possibly reflecting the different data sources used. Guidelines varied in the additional areas that they addressed: potassium, magnesium and calcium supplementation, management of stress and caffeine consumption were considered by some of the guidelines. This demonstrates one of the challenges facing guideline developers. Each clinical care pathway involving assessment, diagnosis, treatment and follow-up requires multiple complex decisions. A clinical guideline will be unable to offer guidance on every consideration that must be made by caregivers and patients. Guidelines will reflect this complexity and are likely to vary in their scope and coverage of the decisions involved in the care pathway.

#### Criteria for initiation of antihypertensive drug therapy

Guidelines used a varying combination of blood pressure and other factors to establish a threshold for drug therapy (see Table 5). These factors included the presence of concomitant disease, target organ damage, cardiovascular risk factors, response to lifestyle changes and the patient's own personal preferences. For uncomplicated patients the relatively recent SA and JNC guidelines recommended lower thresholds. All guidelines (except CMA) recommended lower thresholds for patients with target organ damage, renal disease or diabetes. Some guidelines modified their recommendations for older age groups and specific ethnic groups.

#### First line drug therapies

Variations existed in thresholds for initiating drug treatment and initial drug therapy in typical patients (table 5). Five guidelines recommended the use of thiazdes as initial therapy in non- black patients aged over 55–65 years (SIGN, CMA, ICSI, SA, NICE). The other five differed in their recommendations, one recommended thiazides or b-blockers (VHA), one recommended low dose monotherapy selecting from a broad range of antihypertensive agents (WHO), one recommended low dose monotherapy or a combination of low dose antihypertensive agents (ESH), one recommended thiazides or calcium channel blockers (BHS) and one recommended thiazides alone or in combination with a range of antihypertensive drugs (JNC). The pattern of variation in these recommendations did not follow publication date of the guideline or relate to the research sources used in the development of the guideline.

### Grading recommendations and links to the evidence base

#### Salt intake

Although all guidelines recommended restriction of salt intake, four (SIGN, CMA, BHS, NICE) relied upon a similar and extensive body of work, either directly using the original data in a systematic review or indirectly sourcing the study via a previously published systematic review (see table 6). Nevertheless, these four guidelines were inconsistent in their grading of the recommendation: two guidelines (CMA, NICE) graded it 'B' suggesting that the pattern of care was recommended with caution and based upon research evidence subject to bias, while two graded it as 'A' (BHS, SIGN) indicating that the recommendation was based on strong research evidence not vulnerable to bias. Although the VHA guideline cited much less evidence than these four guidelines, it nevertheless graded salt restriction as 'I', equivalent to 'A' in other schemes. These disparities reflects the differences in the grading of recommendations in guideline development. Both the CMA and NICE guidelines adopted systems that required judgement about the quality of the RCT and the strength of its findings rather than a system that graded recommendations solely upon research design. The other five guidelines made similar recommendations about salt restriction, although they cited very limited evidence to support this.

#### First line drug therapy

Overall the guidelines were relatively consistent in the studies that they cited as the evidence for the drug treatment recommendations (Table 7). One guideline (VHA) cited very little evidence; another (ICSI) did not cite any systematic reviews but referred to recent primary reports of trials, two guidelines (CMA, SA) relied almost exclusively on existing systematic reviews, whereas others (WHO, SIGN, ESH, JNC, BHS) supplemented citation of systematic reviews with citation of recent primary reports; one guideline (NICE) performed its own systematic review. Recommendations for use of thiazides and/or beta-blockers as initial drug therapy in typical patients were graded as 'A' by three guidelines (NICE, SIGN and CMA). One guideline (BHS) recommended thiazides or calcium channel blockers grading it 'C'. This recommendation was largely based on the ABCD algorithm which in turn is based upon an extrapolation of how different drugs work rather than RCT findings; hence the evidence was graded as category III (descriptive studies, or evidence extrapolated from RCTs or quasi experimental studies), leading to the grade of 'C' for the recommendation.

## Discussion

Current guidelines are inconsistent in their handling of key methodologies that relate to the sourcing, interpretation and application of research evidence. Some cite a substantial body of evidence whereas others present little evidence. Some grade their recommendations – although the grading systems and grades used are not consistent – whereas others do not. These findings are consistent with other studies exploring the quality of guideline development [24-27]. Methodological failings may affect the quality of the guideline in several ways. A search that is insufficiently thorough may introduce bias into the summary of the evidence [28]. Systematic reviews have been described as the optimum method of summarising evidence of effectiveness within a clinical practice guideline [29]. In this study we found that most of the guidelines relied on previously published systematic reviews, despite the possible problems with this strategy. Firstly, systematic reviews may date quickly and not incorporate newer evidence. Secondly, the scope of the published reviews may not always match the remit of the guideline and so may not be relevant to the target population of the guideline. Thirdly, up-to-date high quality systematic reviews may not be available in all the areas covered by a guideline.

Despite the inconsistent approach in the guidelines to sourcing the evidence, interpreting it and applying it to recommendations, and the great variation in the volume of supporting evidence cited, the areas of consensus are substantial. Different hypertension guidelines made similar recommendations for many areas of management, notably recommendations for lifestyle changes and their role as first line interventions for patients in certain categories of risk. This level of agreement suggests the possibility either that the published guideline did not cite all the evidence which influenced the recommendations, or that guideline groups may develop guidelines that are heavily influenced by previously published guidelines or an implicit international consensus. Guidelines generated without a systematic search of the literature and without systematic review of all the supporting evidence would be more likely to reflect the biases of developers and it would not be surprising if they were congruent with other guidelines in the same area.

Only five guidelines graded the recommendations made. Failure to grade research evidence and the subsequent recommendations means that the decision making process is not explicit and does not inform the guideline user of the strength of evidence underpinning a particular pattern of care.

The inconsistent grading of the same recommendation in different guidelines also indicates varying approaches to interpreting and applying research evidence in guideline development. The process of evaluating the quality of research evidence and applying this to guideline recommendations using a system of grading is clearly inconsistent and currently an evolving area of guideline methodology.

As well as seeking research evidence, guidelines seek to elicit and incorporate the views of clinical experts and various stakeholders in interpreting the evidence or in offering expert opinion where objective evidence is sparse. Indeed this is an important feature used to assess the quality of a guideline [30]. Differences in guidelines, reflecting the differing views of individuals participating in the guideline development process, are therefore to be expected [29,31]. Herein lies a tension between the rigour needed to try and produce objective and unbiased statements and to also be responsive to the views of participants. It is clear from other reviews of clinical guidelines that the composition of the development group is reflected in the recommendations. Savoie et al [31] in their critical appraisal of guidelines for cholesterol testing found that the greater the involvement of clinical experts in the development process of the guideline, the less the recommendations reflected the research evidence. As only three of the guidelines which we considered fully reported the composition of the guideline development group, it is not possible to make inferences about its impact on recommendations for hypertension. Achieving evidence based guidelines while incorporating the views of the various stakeholders within the development group may create conflict and divergence that the final guideline may mask. The differences between the guidelines described here may reflect this tension.

Differences in recommendations may reflect not only differences in material sourced, differences in interpreting and grading the evidence but also different influences in moving from evidence to recommendations. Differing recommendations for first line drug therapy suggest this. The research base underpinning the recommendations for first line drug therapies is strong in terms of the number and quality of trials in the area (see table 6). Nevertheless, three guidelines (NICE, SIGN and CMA) recommended thiazides for the uncomplicated patient, grading this as 'A', while one (BHS) recommended thiazides or calcium channel blockers grading it 'C'. This suggests either a strong competing interest or the possibility that a less supported but broader recommendation is felt to hold greater clinical merit than older treatments which have accumulated a strong research base. This again may reflect a tension in the development of guidelines, between the restrictiveness of the conventional evidence based approach which inevitably relies on older, well researched therapies and the greater openness of an approach which allows newer, less well endorsed treatments.

Clinical guidelines are rarely based solely on the research evidence and incorporate the consensus views of experts. Raine et al. argue that current approaches to guideline development often lack a sufficient transparency and reliability concerning how such consensus opinions are formed [32]. They highlight the possible influence of key individuals, unrepresentative decision making and the role of constraints of time and resources which limit the range of guidelines that can be generated and their need for updating. They propose an approach which makes reasons for disagreement and degree of consensus explicit and suggests the inclusion of a survey stage to enhance reliability.

The potential influence of external pressures in the formation of guideline recommendations highlights the need for transparency in the declarations of conflictions of interest by authors of clinical practice guidelines. One investigation of panels that write clinical guidelines found that more than one-third of authors declared financial links to relevant drug companies, with around 70% of panels being affected [33]. Another study found that 87% of authors of clinical practice guidelines had some form of interaction with the pharmaceutical industry [7]. If authors have relationships that pose a potential conflict of interest these need appropriate disclosure so that readers may evaluate the merit of those guidelines.

## Conclusion

Many challenges exist to improve the use of evidence in all its forms in guideline development. Clinical practice guidelines remain a developing healthcare technology and if they are to fulfil their potential as a tool to improve standards of care these challenges need to be addressed. The requirements of future guidelines are clear if they are to inform clinicians and patients about appropriate healthcare. Authoritative and rigorously developed guidelines should (where possible) feature transparent and fully reported: guideline group methods and participation; involvement of stakeholders and sponsors; reporting and use of evidence and linking of recommendation to evidence; understanding of health care delivery, the policy context and narratives of patient experience.

## Abbreviations

ACE angiotensin converting enzyme

ARB angiotensin receptor blocker

BMI body mass index

g gram

kg kilogram

m metre

mmHg millimetres of mercury

mins minutes

## Competing interests

The authors contributed to the development of one of the guidelines reviewed [26].

## Authors' contributions

FC, HOD and JMM wrote the manuscript.

ME critically revised the manuscript.

FC, JC, FRB, HOD and JMM performed data abstraction.

FC and FRB performed literature searches.

JMM designed the study.

## Pre-publication history

The pre-publication history for this paper can be accessed here:



## Figures and Tables

**Table 1:** Recent major guidelines for hypertension

**Organisation**	**Publication Year**	**Acronym**
Canadian Medical Association [13]	1999	CMA
World Health Organisation – International Society of Hypertension [14]	1999	WHO
Veterans Health Administration (US) [15]	2000	VHA
Scottish Intercollegiate Guidelines Network [18]	2001	SIGN
European Society of Hypertension[19]	2003	ESH
Institute for Clinical Systems Improvement (US) [16]	2003	ICSI
Joint National Committee VII (US)	2003	JNC
Southern African Hypertension Society [17]	2003	SA
British Hypertension Society [21]	2004	BHS
National Institute for Clinical Excellence [22]	2004	NICE

**Table 2:** Methodological aspects of recent major guidelines for hypertension

**Guideline**	**Development group described^a^**	**New systematic reviewing**	**Used existing systematic reviews^c^**	**Literature search^b^**	**Grading of evidence^d^**	**Grading of recommendations^d^**
CMA	P	N	Y	Y	Y	Y
WHO	P	N	Y	N	N	N
VHA	P	N	N	Y	Y	Y
SIGN	Y	N	Y	Y	Y	Y
ESH	Y	N	Y	N	N	N
ICSI	N	N	y	N	Y	N
JNC	P	N	Y	N	Y	N
SA	P	N	y	N	N	N
BHS	P	N	Y	N	Y	Y
NICE	Y	Y	Y	Y	Y	Y

a Y/N: Includes/does not include description of stakeholders involved in the development process, including patient representatives and any conflicts of interest.

P: Partially – a list of names with institutional affiliations was provided.

b Y/N: Searches were/were not undertaken, at least through Medline.

c Y: Made extensive use of existing systematic reviews (referenced 7 or more).

y: Made partial use of existing systematic reviews (referenced 6 or less).

N: Did not use existing systematic reviews.

d Y/N: Reports/does not report explicit grading of the quality of the supporting evidence/recommendations.

**Table 3:** Methodological aspects of previously published guidelines

**Guideline**	**Literature search**	**Grading of evidence**	**Development group**
**CMA**	MEDLINE and Cochrane Collaboration searches; reference lists in retrieved articles. Requests to experts and panel members.	Recommendations were graded from A-D and were based on assessment of the studies using an algorithm. A Grade assigned if recommendation was: – based on an adequate RCT ie, with blinded assessment of outcomes, intention-to-treat analysis, adequate follow-up, and sufficient sample size to detect a clinically important difference with power greater than 80%. – based on an adequate subgroup analysis – based on a systematic review in which the comparison arms are derived from head-to head comparisons with the same RCT	A committee with a range of representatives from different bodies. Patient involvement unclear

**WHO**	None described	None described	Patient involvement unclear

**VHA**	MeSH terms covering key therapies, and study characteristics and design	Evidence was graded: A RCT B well-designed clinical studies C panel consensus Recommendations were rated: I usually indicated; always acceptable; useful and effective IIa acceptable, of uncertain effectiveness, and may be controversial. Weight of evidence in favour of usefulness/effectiveness IIb acceptable, of uncertain effectiveness and may be controversial. Not well established by evidence, can be helpful and probably not harmful	Thirty-eight individuals. Roles not always clear.

**SIGN**	Systematic literature searches on MEDLINE, Healthstar, EMBASE, Cochrane Library. Based on a published Cochrane review	Evidence was graded when obtained from: Ia: meta-analysis of RCTs Ib: at least 1 RCT IIa: at least 1 well-designed controlled study without randomisation IIb: at least one other type of well-designed quasi-experimental study III: well-designed non-experimental descriptive studies IV: expert committee reports and/or respected clinical opinion Recommendations were rated: A Evidence levels Ia, Ib B Evidence levels IIa, IIb, III C Evidence level IV.	Members' names and affiliations listed and conflicts of interest available. Specialist reviewer names given. Age Concern represented.

**ESH**	None described	Recommendations not classified upon strength of available evidence.	Members' names, affiliations, potential conflicts of interest given. Patient involvement unclear

**ICSI**	None described	Research reports were graded as follows: Primary reports A (RCT) to D (case and cross sectional studies) Reviews M (Meta-analysis, systematic reviews, decision analysis, cost-benefit analysis, cost-effectiveness study) R: Narrative review, consensus statement or report X: Medical opinion In the 2002 update, some recommendations link to the evidence grade	No details

**JNC**	None described	Evidence supporting recommendations for prevention and treatment was classified: M meta-analysis Re retrospective analyses (case control) RA RCT F prospective follow-up – cohort study Pr previous review C clinical interventions (non-randomised) X cross-sectional population studies (prevalence)	Nine individuals. Contributions were sought from multidisciplinary experts. No mention of patient involvement

**SA**	None described	Evidence not described or graded	Members' names and affiliation given. Patient involvement unclear

**BHS**	Not described	Strength of evidence: Ia (meta analysis of RCTs) to IV expert opinion Strength of recommendation A Directly based on category I evidence B Directly based on category II evidence or extrapolated recommendation from category I evidence C Directly based on category III evidence or extrapolated recommendation from category I or II evidence. D Directly based on category IV evidence or extrapolated recommendation from category I, II or III evidence	No details given

**NICE**	Search using MEDLINE, EMBASE and CENTRAL, previous systematic reviews, bibliographic seachs as well as contact with subject area experts.	Guideline Recommendation and Evidence Grading (GREG [34]) system applied. **Evidence Grade: Interpretation of Evidence** I High Plausible, precisely quantified and not vulnerable to bias. II Intermediate Plausible but not quantified precisely or may be vulnerable to bias. III Low Concerns about plausibility or vulnerability to bias. **Recommendation Grade Interpretation of recommendation** A Recommendation Robust evidence B Provisional Recommendation Recommend with caution C Consensus Opinion Recommended by consensus	Members names and affiliations given and any potential conflicts of interest. Contributions were sought from multidisciplinary experts. Group included patient representatives

**Table 4:** Recommendations for lifestyle interventions in recent major guidelines for hypertension

**Guideline**	**Weight reduction**	**Diet**			**Salt restriction**	**Alcohol restriction**	**Smoking cessation**	**Exercise**
						
	*Recommended weight*	*Reduce saturated fat*	*Increase fruit & vegetables*	*Other recommendations*	*Recommended max. daily intake*	*Recommended max. daily intake (ethanol)*		
CMA	Y (BMI 20–25 kg/m^2^)	No specific recommendation			Y (3–7 g/day)	≤17.4 g/day (women) ≤27.0 g/day (men)	Y	50–60 mins. aerobic exercise 3–4 times per week
WHO	Y if overweight	Y	Y	Increase fish consumption	Y (≤ 6 g/day)	≤10–20 g/day (women) ≤ 20–30 g/day (men)	Y	30–45 mins. aerobic exercise 3–4 times /week
VHA	Y (if > 10% of ideal body weight)	Y	Y	Increase cereal consumption	Y (≤ 6 g/day)	≤14 g/day (women) ≤ 28 g/day (men)	Y	30–45 mins. aerobic exercise 3–5 times/week
SIGN	Y (BMI < 25 kg/m^2^)	Y	Y		Y (<5 g/day)	≤15.8 g/day (women) ≤ 23.7 g/day (men)	Y	30–45 mins. aerobic exercise most days
ESH	Y	Y	Y	Increase fish consumption	Y (4.7–5.8 g/day)	≤10–20 g/day (women) ≤ 20–30 g/day (men)	Y	30–45 mins. aerobic exercise 3–5 times/week
ICSI	Y if overweight	Y	Y	Increase low fat dairy food consumption	Y (<6 g/day)	≤14 g/day (women) ≤ 28 g/day (men)	Y	30–45 mins. aerobic exercise 3–4 times/week
JNC	Y (BMI 18.5–25 kg/m^2^)	Y	Y	Increase low fat dairy food consumption	Y (≤ 6 g/day)	≤14 g/day (women) ≤ 28 g/day (men)	Not discussed	≥30 mins. aerobic exercise most days
SA	Y (BMI < 25 kg/m^2^)	Y	Y	Increase fibre, unrefined carbohydrates	Y	≤24 g/day	Y	30 mins. aerobic exercise 3–5 times per week
BHS	Y (BMI 20–25 kg/m^2^)	Y	Y	Reduce total fat	Y (< 6 g/day)	≤15.8 g/day ≤ 23.7 g/day (men)	Y	> 30 mins. aerobic exercise most days
NICE	Y	Healthy, low calorie diet			Y (<6 g/day)	< 14 units/wk (women) < 21 units/week (men)	Y	30–60 mins. 3–5 times/week

**Table 5:** Recommended thresholds for drug treatment and initial drug therapy in recent major guidelines for hypertension

	Thresholds for initiating drug treatment*				**Initial drug therapy in uncomplicated patient (non-black patient aged over 55–60 years)†**	
*Guideline*	*No target organ damage or risk factors*	*With risk factors (other than diabetes mellitus)*	*With target organ damage*	*With diabetes mellitus or renal disease*	*Recommendation*	*Grade of recommendation*

CMA	≥160/90	≥160/90	≥160/90	≥140/90	Thiazides	A
WHO	≥150/95	≥140/90	≥140/90	≥130/85	Low dose monotherapy	
VHA	>160 and/or>100	>160 and/or >100	≥130/85	≥130/85	Thiazides or β-blockers	
SIGN	≥160/100	≥160/100	≥140/90	≥140/90	Thiazides	A
ESH	≥150/95	≥140/90	≥130/85	≥130/85	Low dose monotherapy or a combination	
ICSI	≥160/100	≥140/90	≥130/85	≥130/85	Thiazides	
JNC	≥140/90	≥140/90	≥130/80	≥130/80	Thiazides alone and/or combination ACE, ARB, β-blockers, CCB	
SA	≥140/90	≥140/90	≥130/85	≥130/85	Thiazides	
BHS	≥160/100	≥140/90	≥140/90	≥140/90	Thiazides or calcium channel blockers	C
NICE	≥160/100	≥160/100 or ≥ 140/90 if CHD^§ ^risk ≥ 15% or CVD^¶^risk ≥ 20%	≥140/90	Outside scope of guideline	Thiazides	A[34]

* Blood pressure threshold in mmHg

† Thiazides: low dose thiazide diuretics

ACE: angiotensin-converting enzyme inhibitors

ARB: angiotensin receptor blockers

CCB: calcium channel blocker

§CVD: cardiovascular disease

¶CHD: coronary heart disease

**Table 6:** Reporting in guidelines of trials and systematic reviews of salt reduction

**Reference**	**Year**	**CMA **1999	**WHO **1999	**VHA **1999	**SIGN **2001	**ESH **2003	**ICSI **2003	**JNC **2003	**SA **2003	**BHS **2004	**NICE **2004
Randomised Controlled Trials											
Silman [35]	1983	Y								SR[36]	Y
Fagerberg [37]	1985	SR[38]			SR[38]					SR[38]	Y
Chalmers [39]	1986	SR[38]	SR[40]		SR[38]					SR[38]	Y
Chalmers [41,42]	1989					SR[40]					Y
TOHPI	1992			Y							Y(ex)
Jula [43]	1994	SR[38]			SR[38]					SR[38]	Y
Whelton [44]	1998				Y					Y	Y(ex)
Appel [45]	2001						Y				Y(ex)
Sacks [46]	2001							Y	Y		Y(ex)
Systematic Reviews											
Law [47]	1991			Y			Y				Y
Midgely [38]	1996	Y			Y					Y	Y
Cutler [40]	1997		Y			Y					Y
Hooper [36]	2002									Y	Y
Grade of recommendation											
		B		I	A	-	-	-	-	A	B[34]

Y Trial cited by guideline

SR Trial included in systematic review cited by guideline

Y(ex) Trial cited by guideline but excluded from meta-analysis

**Table 7:** Reporting in guidelines of trials of antihypertensive medication ^a^

**Trial^a^**	**Year^b^**	**CMA 1999**	**WHO 1999**	**VHA 1999**	**SIGN 2001**	**ESH 2003**	**ICSI 2003**	**JNC 2003**	**SA 2003**	**BHS 2004**	**NICE 2004**
VA-II[48]	1970	SR[49, 50]	SR[49,51]		SR[49,51,52]	SR[51]		SR[53]	SR[51]	SR[49]	
HSCSG[54]	1974	SR[50]	SR[51]		SR[51]	SR[51]		SR[53]	SR[51]		
USPHS[55]	1977	SR[50]	SR[51]		SR[51]	SR[51]		SR[53]	SR[51]		
VA-NHLBI[56]	1978	SR[50]	SR[51]		SR[51]	SR[51]		SR[53]	SR[51]		
HDFP[57-59]	1979	SR[49]	SR[49,51]		SR[49,51,52,60]	SR[51,61]			SR[51]	SR[49]	
ANBPS[62]	1980		SR[51]		SR[51,52,60]	SR[51,63]		SR	SR[51]		
EWPHE[64,65]	1985	SR[49,50]	SR[49,51,66]			SR[51,61]		SR[53]	SR[51]	SR[49]	
IPPPSH[67]	1985										
MRC[68]	1985	SR[50]			SR[51]			SR[53]	SR[51]		
Coope[69]	1986	SR[49]	SR[49,51,66]			SR[51,61]		SR[53]	SR[51]	SR[49]	
OSLO[70]	1986	SR[50]	SR[51]		SR[51]	SR[51]		SR[53]	SR[51]		
SHEP-P[71-73]	1986	SR[50]			SR[60]	SR[61]		SR[53]			
HAPPHY[74]	1987	SR[50]									
MAPHY[75]	1988										
CAPPP[76,77]	1990										
SHEP[78-81]	1991										
STOP-H[82]	1991	SR[49]				SR[61]		SR[53]		SR[49]	
SYST-EUR[83-86]	1991					SR[87]					
MRC-O[88]	1992	SR[50]				SR[61]		SR[53]		SR[49]	
STOP-H2[89-91]	1993	SR[92]				SR[63,87,93]					
NICS-EH[94,95]	1994	SR[92]				SR[63,87,93]				SR[63,96]	
HOT[97]	1995										
PATS[98]	1995										
ALLHAT[99,100]	1996										
MIDAS[101,102]	1996	SR[50,92]								SR[63]	
VHAS[103]	1997	SR[50,92]				SR[3,87,93]				SR[63,96]	
ABCD[104]	1998	SR[92]				SR[87]					
FACET[105]	1998	SR[92]									
UKPDS[106]	1998										
HOPE[107]	2000										
INSIGHT[108,109]	2000	SR[92]									
NORDIL[110]	2000	SR[92]									
IDNT[111]	2001					SR[93]				SR[96]	
PROGRESS[112]	2001		c								
RENAAL[113]	2001										
ELSA[114]	2002					SR[93]				SR[96]	
LIFE[115]	2002										
ANBP2[116,117]	2003					SR[93]				SR[96]	
CONVINCE[118,119]	2003									SR[96]	
SCOPE[120]	2003									SR[96]	
JMIC-B[121]	2004									SR[96]	
VALUE[122-124]	2004										

a Trials post-dating the guidelines (indicated by the stepped line) were not available to the guideline developers.

b Year of earliest publication.

c Referred only to rationale and design.

Primary report of trial cited by guideline.

SR Trial included in systematic review cited by guideline; primary report not cited by guideline.
